# Induction chemotherapy with concurrent chemoradiotherapy versus concurrent chemoradiotherapy for locally advanced squamous cell carcinoma of head and neck: a meta-analysis

**DOI:** 10.1038/srep10798

**Published:** 2015-06-04

**Authors:** Lijuan Zhang, Nan Jiang, Yuexian Shi, Shipeng Li, Peiguo Wang, Yue Zhao

**Affiliations:** 1 School of Nursing, Tianjin Medical University, Tianjin 300070, China; 2 First Central Clinical College of Tianjin Medical University, Tianjin 300192, China; 3 Department of Radiotherapy, Tianjin Medical University Cancer Institute and Hospital, National Clinical Research Center for Cancer, Tianjin 300060, China

## Abstract

Concurrent chemoradiotherapy (CCRT) has been considered to be the standard of care for locally advanced squamous cell carcinoma of head and neck (LA-SCCHN). Whether induction chemotherapy (IC) with CCRT will further improve the clinical outcomes or not is still unclear. We conducted a meta-analysis to compare the two regimens for LA-SCCHN. Literature searches were carried out in PubMed, Embase, Cochrane Library and Chinese Biology Medicine from inception to November 2014. Five prospective randomized controlled trials (RCTs) with 922 patients were included in meta-analysis. Results were expressed as hazard ratios (HRs) or relative risks (RRs) with 95% confidence intervals (CIs). Compared with CCRT, IC with CCRT showed no statistically significant differences in overall survival (OS), progression-free survival (PFS), overall response rate (ORR) or locoregional recurrence rate (LRR), but could increase risks of grade 3–4 febrile neutropenia (P = 0.0009) and leukopenia (P = 0.04). In contrast, distant metastasis rate (DMR) decreased (P = 0.006) and complete response rate (CR) improved (P = 0.010) for IC with CCRT. In conclusion, the current studies do not support the use of IC with CCRT over CCRT, and the further positioning of IC with CCRT as standard treatment for LA-SCCHN will come from more RCTs directly comparing IC followed by CCRT with CCRT.

Head and neck cancer is the sixth most common type of cancer, representing about 6% of all cases and accounting for an estimated 650,000 new cancer cases and 350,000 cancer deaths worldwide every year^1^. Almost all of these malignancies originating from the mucosal epithelia are squamous cell carcinoma of the head and neck (SCCHN)^2^. SCCHN typically benefits from single modality therapy of either surgery or radiotherapy (RT), with excellent disease control and long-term survival when diagnosed at an early stage (stage I/II). The 5-year overall survival (OS) rate is 80% to 90% for a stage I disease and 65% to 80% for a stage II^3^,^4^,^5^,^6^. Unfortunately, most patients are present with stage III or IV disease when diagnosed^7^. For those patients with locally advanced SCCHN (stage III/IV, LA-SCCHN), the prognosis is quite poor, and 40%–60% of patients relapse and 30%–50% of patients live for 3 years after treatment with surgery and radiotherapy (RT)^8^,^9^. Given that, the comprehensive, sequential, multi modality treatment regimens play an important role in the whole treatment^10^. Several large, randomized controlled trials have demonstrated the superiority of a combined modality treatment regimen^11^,^12^,^13^. These results were confirmed by a recent meta-analysis on 93 randomized trials and 17,346 patients, demonstrating an absolute survival benefit for the addition of chemotherapy of 4.5% at 5 years, regardless of the sequence used (adjuvant, induction or concomitant)^14^. Concurrent chemoradiotherapy (CCRT) seemed to be considered the standard management for LA-SCCHN in many centers^11^,^15^,^16^. However, this regimen was challenged by the introduction of induction chemotherapy (IC), which produced response rates of 80–90%, with complete response rates of 20–40% in LA-SCCHN^17^. In this case, several studies aimed to intensify the treatment regimen by adding IC before CCRT. IC followed by CCRT is also known as sequential chemoradiotherapy. One small randomized phase II study demonstrated that patients with LA-SCCHN had a benefit for sequential chemoradiotherapy in terms of radiologic complete response rates (CR)^18^. However, the others failed to demonstrate a survival benefit following addition of IC to primary CCRT^19^,^20^,^21^,^22^.

IC followed by CCRT is often used in patients with LA-SCCHN despite no clear evidence exists in prolonging survival for those patients with LA-SCCHN. Against this background, we present a meta-analysis to evaluate the efficacy and toxicity of IC followed by CCRT versus CCRT alone in the treatment of LA-SCCHN.

## Results

### Characteristics of included trials and patients

**Figure 1:**
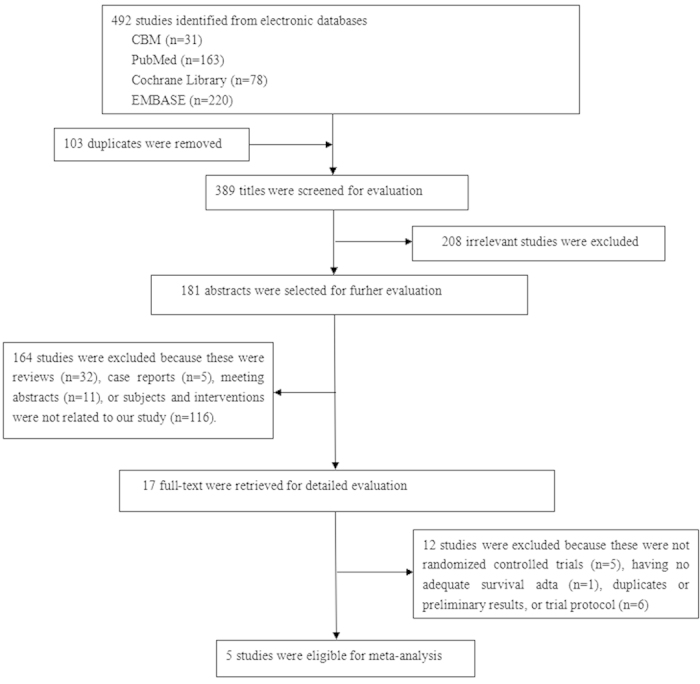


A total of 492 trials were screened for eligibility, of which 103 were duplicates. After screening the titles of these trials, we excluded 208 irrelevant trials. Then 181 abstracts were selected for further evaluation, of which 164 trials were excluded because these were reviews, case reports, meeting abstracts or subjects and interventions were not related to our study. After review of the full texts, five prospective randomized controlled trials (RCTs) met the inclusion criteria and were included in meta-analysis^18^,^19^,^20^,^21^,^22^. One trial was presented at 2014 American Society of Clinical Oncology (ASCO) Annual Meeting related to our study after reviewing the abstract^23^, but the full text was not available from the databases. We sent an e-mail to the author but no reply was received. Finally, five RCTs were included in our meta-analysis. Selecting process was showed in Fig. 1. All included trials compared IC followed by CCRT with CCRT alone for LA-SCCHN (III-IVM0).

The baseline characteristics of these trials were shown in Table 1. In total, our analysis included 922 patients, 473 patients in IC followed by CCRT arm, and 449 patients in CCRT alone arm. The rate of complete treatment in IC followed by CCRT arm ranged from 80% to 92% and in CCRT arm the rate was 84%–96%. Every trial reported hazard ratios (HRs) with 95% confidence intervals (CIs) for overall survival (OS) and/or progression-free survival (PFS), or data to calculate these. These trials were published between 2010 and 2014. All trials were RCTs. The sample size ranged from 101 patients^18^ to 283 patients^22^. One trial in Italy^18^, one in China^19^, two trials were undertaken in USA^20^,^21^, and one in Spain^22^. Four trials were multicenter studies^18^,^20^,^21^,^22^, and one was single-institution study^19^. Most of the reported patients were male, accounting for 86.3%. All trials provided data on the mean/median age of patients, and the lowest and highest mean/median ages were 54 and 60 years. Total radiation dose ranged from 66 Gy to 75 Gy. For IC, TPF regimen was given as docetaxel or paclitaxel plus cisplatin and fluorouracil, expect for Chen *et al.* trial^19^, the IC regimen did not contain fluorouracil. Cycles were repeated every 3 weeks for 3 or 2 cycles. However, for CCRT regimen, there were some differences among the five trials. Cisplatin plus 5-fluorouracil or paclitaxel^18^,^19^, single agent docetaxel, carboplatin or cisplatin^20^,^22^; hydroxyurea, fluorouracil and docetaxel combination^21^.

### Quality assessment

The results of quality assessment were shown in Table 2. Among five RCTs, three trials were assigned in B level^18^,^19^,^22^, whereas two trials had a high risk of allocation concealment and blinding 20,21, so we rated them in C level. All of the five trials included in this study could be identified as having adequate random sequence generation, and the trials addressed incomplete outcome data and selective reporting. There was only one trial that used allocation concealment^18^. Almost all trials did not use blinding, expect for one trial reported the blinding of outcome assessment^18^. However, one trial was halted because of slow accrual, which prompted us the existence of other bias^20^.

### Efficacy of IC followed by CCRT versus CCRT alone

**Figure 2:**
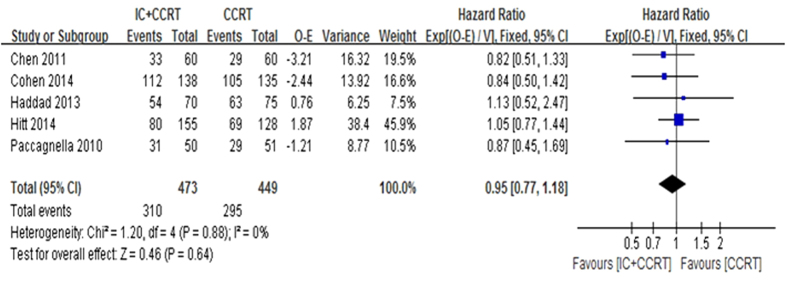


**Figure 3:**
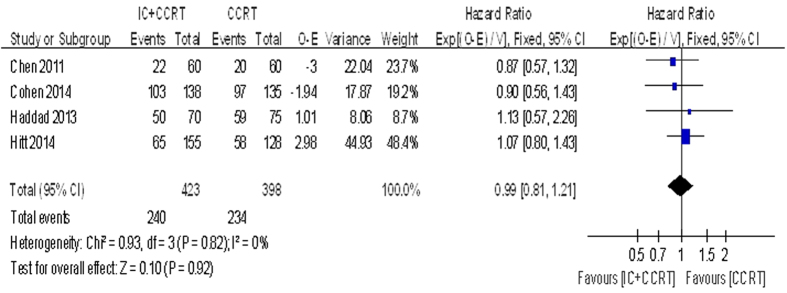


The 2-year OS was based on the five trials with 922 patients for meta-analysis. Data of trials, such as HR, 95% CI, O-E and its variance were extracted by indirect methods. Unfortunately, meta-analysis showed that no significant beneficial effect was observed for 2-year OS (HR = 0.95, 95% CI 0.77–1.18, P = 0.64. Fig. 2). There was no statistically significant heterogeneity among the trials (I^2^ = 0%, P = 0.88). The 3-year OS (HR = 0.99, 95% CI 0.81–1.21, P = 0.92. Fig. 3) and the 5-year OS (HR = 1.00, 95% CI 0.82–1.20, P = 0.9) were based on the four trials with 821 patients for meta-analysis. There also no significant beneficial effect was showed, and no statistically significant heterogeneity existed.

**Figure 4:**
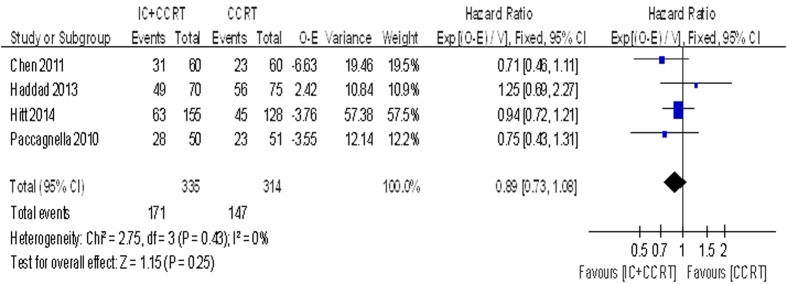


Data regarding the 2-year PFS was reported in four trials with 649 patients. We observed no great benefit of IC followed by CCRT comparing with CCRT alone (HR = 0.89, 95% CI 0.73–1.08, P = 0.25. Fig. 4). The 3-year PFS (HR = 0.92, 95% CI 0.75–1.12, P = 0.41) and the 5-year PFS (HR = 0.91, 95% CI 0.75–1.11, P = 0.36) were based on the three trials with 548 patients for meta-analysis. No significant beneficial effect was showed, and no statistically significant heterogeneity existed.

**Figure 5:**
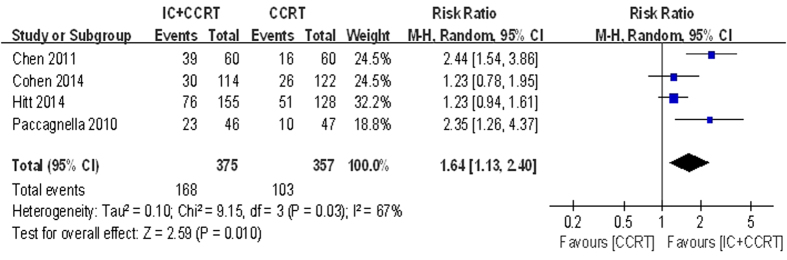


Data regarding the Post-CCRT of overall response rate (ORR) and complete response (CR) were available in four trials with 732 patients. IC followed by CCRT compared with CCRT alone had no statistically significant effect on ORR (RR = 1.03, 95% CI 0.95–1.11, P = 0.49) but not for the CR (RR = 1.64, 95% CI 1.13–2.40, P = 0.010. Fig. 5). However, there was heterogeneity in the CR of the effect across the included studies (I^2^ = 67%, P = 0.03), so we carried out the data analysis by the random-effects model.

**Figure 6:**
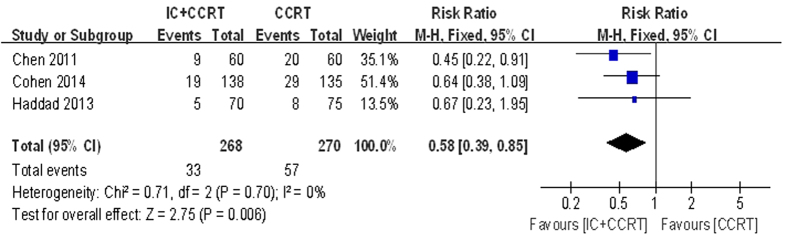


No significant difference was found in OS comparing the patients receiving IC followed by CCRT with those receiving CCRT alone, but the patients receiving IC followed by CCRT had a significantly lower distant metastasis rate (DMR) with a benefit of relative risks (RR) 0.58 ( 95% CI 0.39–0.85, P = 0.006. Fig. 6). In contrast, the locoregional recurrence rate (LRR) was not significantly different between these two arms (RR = 1.10, 95% CI 0.82–1.47, P = 0.54). There was no statistically significant heterogeneity among the trials.

### Toxicity of IC followed by CCRT versus CCRT alone

Only two trials involving 289 patients provided information on adverse events during IC period^21^,^22^, the most commonly recorded grade 3–4 adverse event was leukopenia accounting for 21.45%, with the next most common event being febrile neutropenia (16.99%). Neutropenia and mucositis accounted for 15.22% and 12.11% respectively (Table 3). The incidences of other toxic events were lower than above findings.

**Figure 7:**
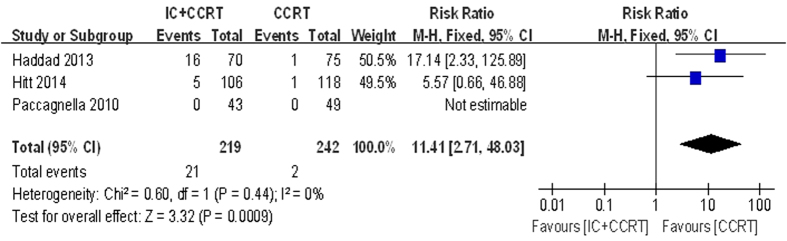


**Figure 8:**
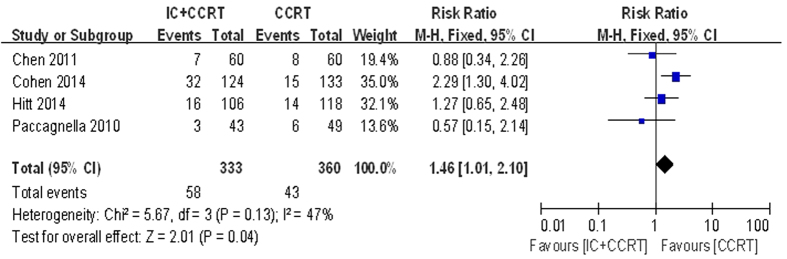


During CCRT period, five haematologic and seven non-haematologic toxic effects were analysed (Table 4). Data for mucositis was available for all included trials. Data for five of twelve analysed (anemia, thrombocytopenia, neutropenia, leukopenia and nausea/vomiting) were available for four trials. Data for febrile neutropenia, fatigue, dermatitis, pain, dysphagia and weight loss were available for three trials. According to the pooled analysis for toxic effects, adding IC before CCRT may increased the risk of grade 3–4 toxic events of febrile neutropenia (RR = 11.41, 95% CI 2.71–48.03, P = 0.0009) and leukopenia (RR = 1.46, 95% CI 1.01–2.10, P = 0.04), compared with CCRT alone (Fig. 7 and Fig. 8). However, the grade 3–4 mucositis (RR = 1.30, 95% CI 0.86–1.97, P = 0.22) had no statistical significance between the two arms. The incidences of each arm were 58.81% and 47.36%. There was no significant heterogeneity between the trials in the toxicity analysis for febrile neutropenia and leukopenia. The incidences of other toxic events were comparable between the two arms.

### Sensitivity analysis

**Figure 9:**
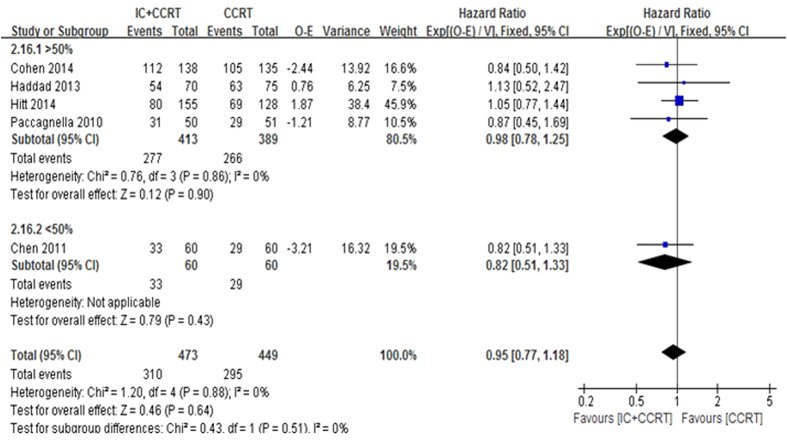


**Figure 10:**
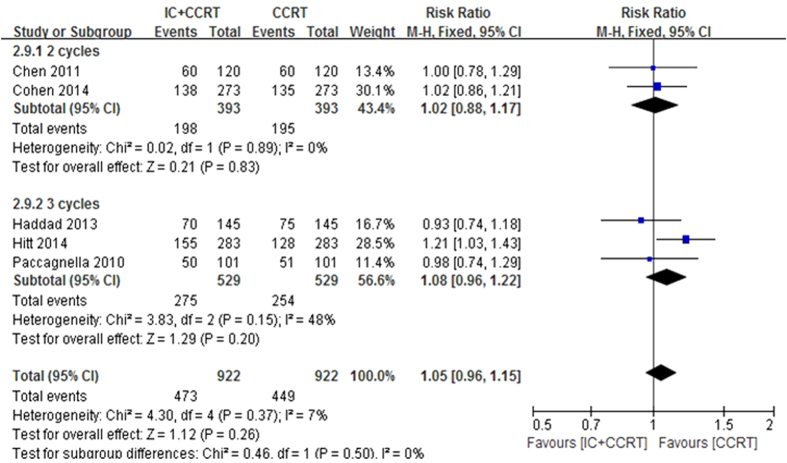


Sensitivity analysis was executed by excluding Chen *et al.* trial^19^ with a different IC regimen using paclitaxel 135–150 mg/m^2^ day1 plus cisplatin 75–100 mg/m^2^ day1 (every 3 weeks for 2 cycles) or patients coming from Asia rather than Europe, and also analysed all included trials^18^,^19^,^20^,^21^,^22^ by 2-year OS >50% versus 2-year OS <50% in the control arm and 2 cycles IC versus 3 cycles IC in the experimental arm to explore the robustness of the result (Fig. 9 and Fig. 10). The results showed no effects on the results irrespective of effect models. No other sensitivity analysis changed our results.

### Funnel plot of publication bias

**Figure 11:**
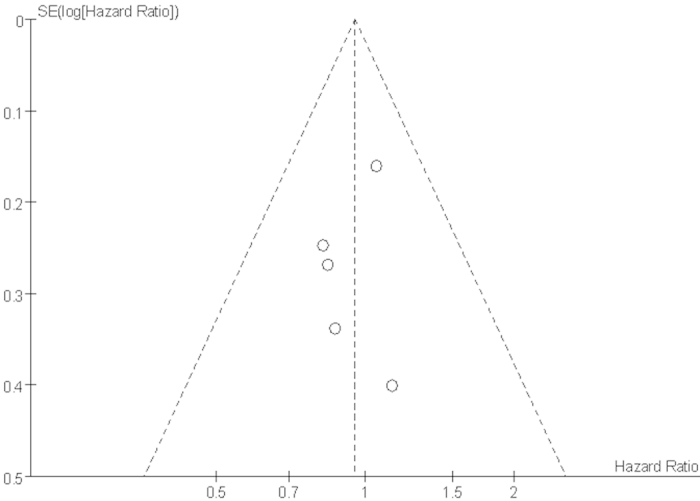


A funnel plot of trials was performed to assess the possibility of publication bias (Fig. 11), which showed asymmetry, thereby indicating that publication bias possibly existed in the included trials.

## Discussion

The treatment paradigm for LA-SCCHN has evolved over the past several decades, but the multidisciplinary approach for the management of LA-SCCHN is still controversially discussed among clinicians. Presently, the treatment of choice for nonresectable SCCHN is CCRT, and this recommendation is based on level I, A grade of evidence according to international guidelines^24^. However, the main benefit of the systemic therapy delivered with CCRT is an improvement of locoregional control, not sterilization of micrometastases^25^. The concept of IC followed by radiotherapy or CCRT has become progressively more popular in an attempt to improve distant disease control^26^. This regimen is practically used in clinical treatment of patients with LA-SCCHN. However, no clear evidence exists in prolonging survival and benefits compared with the present standard CCRT for those patients with LA-SCCHN. Against this background, we present a meta-analysis to evaluate the efficacy and toxicity of IC followed by CCRT versus CCRT alone in the treatment of LA-SCCHN.

We identified five RCTs that evaluated the efficacy and toxicity of IC followed by CCRT versus CCRT alone for LA-SCCHN including Europe and Asia. To the best of our knowledge, this is the first meta-analysis of IC followed by CCRT in comparison to CCRT alone including Europe and Asia 18,19,20,21,22. We comprehensively searched literature regardless of published year and language. Our meta-analysis revealed no significant treatment effect in terms of OS, PFS, ORR and LRR for IC followed by CCRT versus CCRT alone, but could increase risks of grade 3–4 febrile neutropenia (RR = 11.41, 95% CI 2.71–48.03, P = 0.0009) and leukopenia (RR = 1.46, 95% CI 1.01–2.10, P = 0.04). In contrast, IC followed by CCRT could decrease the incidences of DMR (RR = 0.58, 95% CI 0.39–0.89, P = 0.006) and improve the rates of CR (RR = 1.64, 95% CI 1.13–2.40, P = 0.010).

In our meta-analysis, the rate of complete treatment in IC followed by CCRT arm ranged from 80% to 92% and in CCRT arm the rate was 84%–96%. Notably, each of the two regimens was feasible and well tolerated and did not compromise the delivery of treatment plan. However, the analysis failed to show any improvements in terms of OS and PFS for IC followed by CCRT comparing with CCRT alone. The large, well-conducted meta-analysis carried out by Pignon *et al.* in 2000^27^ and then updated in 2009^14^ have confirmed the concomitant use of chemotherapy and radiation to be more successful. A 6.5% 5-year absolute survival benefit (HR = 0.81, 95% CI 0.78–0.86, P < 0.001) was demonstrated for concomitant treatment in their updated individual patient analysis of 17,346 patients from 93 randomized trials^14^. However, no OS benefit was identified from the IC regimen^14^. For several decades, the optimal sequencing of chemotherapy, radiation, and surgery in the management of LA-SCCHN has remained a subject of intense debate. The TAX 323^28^, TAX 324^29^and the GORTEC laryngea^30^ studies investigated the important question of identifying the optimal induction chemotherapy regimen to use in head and neck cancer, which showed that TPF (three-drug taxane, fluorouracil, and cisplatin combination) was significantly better than PF (fluorouracil and cisplatin doublet) for survival, local control, and organ preservation. These studies defined a new standard of care for induction chemotherapy in the USA and Europe, and also led to regulatory approval of TPF for patients with resectable and unresectable disease^20^. Induction (neoadjuvant) chemotherapy practically used in clinical treatment for LA-SCCHN, but the benefit of IC followed by CCRT compared with CCRT alone in the management of LA-SCCHN still remained controversial. To date, five RCTs^18^,^19^,^20^,^21^,^22^ have addressed this question; no survival benefit was derived from what proved to be the more toxic sequential treatment schedule. In accordance with the meta-analysis^31^, the difference of OS and PFS was not significant between the patients receiving IC protocol of TPF (docetaxel/ cisplatin/5-fluorouracil) followed by CCRT and those receiving CCRT alone (331 patients in two trials; HR = 0.96, 95% CI 0.71–1.30, P = 0.78 for OS; HR = 0.99, 95%CI 0.53–1.87, P = 0.98 for PFS). Therefore, there was still not clear evidence that TPF IC regimen improved survival in the patients with LA-SCCHN compared to patients receiving CCRT alone.

According to our meta-analysis, adding IC before CCRT could decrease the DMR (RR = 0.58, 95% CI 0.39–0.85, P = 0.006), but had no statistically significant effect on LRR. Additionally, meta-analysis conducted by Ma *et al.*^31^ and Su *et al.*^32^ had showed that induction (neoadjuvant) chemotherapy had the potential to reduce the incidence of distant metastases, but had no effect on locoregional relapse. Another meta-analysis also had proved IC significantly decreased the DMR (HR = 0.73, 95% CI 0.61–0.88, P < 0.001) but not for locoregional failure^14^. Above all, the IC followed by CCRT regimen has clear benefit of DMR. With regards to DMR, our analysis was highly in accordance with others^14^,^31^,^32^. This again proved the significant effect of IC followed by CCRT on decreasing the incidence of distant metastases.

Data regarding the Post-CCRT of ORR and CR were available in four trials with 732 patients. Our meta-analysis revealed that patients in IC followed by CCRT arm with higher CR (RR = 1.64, 95% CI 1.13–2.40, P = 0.010) compared with CCRT alone. More patients in the TPF group proceeded to CCRT, likely reflecting the higher response rates^29^,^30^,^33^. However, IC followed by CCRT arm was tested no benefit of improving ORR (RR = 1.03, 95% CI 0.95–1.11, P = 0.49). New trials that compare IC plus CCRT versus CCRT alone are further needed to better define the role of IC treatment. It was noted that the trial by Paccagnella *et al.*^18^ was a phase II study, but it fit the including criteria of our meta-analysis. The study directly compared IC followed by CCRT to CCRT alone. Cisplatin and 5-FU were used as radiosensitizer during the CCRT period. The CR rates were 51% in the IC followed by CCRT versus 21% in the CCRT arm. What’s more, IC followed by CCRT regimen was associated with no negative impact on CCRT feasibility. SCCHN is a highly responsive malignancy at initial presentation. Cisplatin-based induction chemotherapy had produced response rates of 80–90%, with CR rates of 20–40% in LA-SCCHN^17^. Despite high antitumour activity, the studies failed to show survival benefits. Previous reports have indicated that patients with CR and with pathologic response to induction chemotherapy have better survival than patients with response to treatment that was less than CR^34^. There was heterogeneity in the CR of the effect across the included studies (I^2^ = 67%, P = 0.03), so we carried out sensitivity analysis by excluding Chen *et al.* trial^19^ which adopted different IC regimen and included different regions of the patients with others. No other sensitivity analysis changed our results.

With regards to our meta-analysis, we noted no differences in non-haematologic toxic effects between the two arms, but in haematologic toxic effects, the grade 3–4 febrile neutropenia (RR = 11.41, 95% CI 2.71–48.03, P = 0.0009) and leukopenia (RR = 1.46, 95% CI 1.01–2.10, P = 0.04) differed. These severe toxic effects were major risk factors for infection-related morbidity and mortality and also a significant dose-limiting toxicity in cancer treatment, which might impact the success of treatment, particularly when treatment intent was either curative or to prolong survival^35^. It indicated that patients treated with CCRT might increase the incidences of adverse events. The grade 3–4 neutropenia had no significant differences between two arms, which might be associated with prophylactic granulocyte colony-stimulating factor (G-CSF)^35^. G-CSF, a hematopoietic cytokine, regulates the proliferation and differentiation of granulocytic progenitor cells and functionally activated mature neutrophils. G-CSF also affects nonhematopoietic tumor cells through its binding to the specific receptor (G-CSFR) on the cells^36^. A meta-analysis carried out by Ma *et al.* provided information that the patients receiving IC followed by RT compared to patients receiving CCRT had a significantly lower occurrence on grade 3–4 mucositis (850 patients in four trials; OR = 0.56, 95% CI 0.40–0.77, P = 0.0005; RR = 0.66, 95% CI 0.52–0.83, P = 0.0005)^31^. However, in our meta-analysis, the grade 3–4 mucositis (RR = 1.30, 95% CI 0.86–1.97, P = 0.22) had no statistical significance between the two arms. Both arms were with similar rates about the grade 3–4 mucositis during CCRT. At present, there is no radioprotectant with proven efficacy in decreasing the severity of mucositis during chemoradiotherapy for SCCHN, and choice of formulation remains a matter for individual clinical judgement^2^,^33^.

Several limitations were presented in this meta-analysis. Firstly, in common with the other published meta-analysis^31^,^32^,^37^, our meta-analysis was based on summary data, and lack of individual patient data preventing us from adjusting treatment effects according to disease and patient variables. Secondly, we had not carried out a comprehensive subgroup analysis because of limited number of the included trials. Thirdly, RT techniques, human papilloma virus (HPV) status^38^,^39^ and prophylactic granulocyte colony-stimulating factor (G-CSF)^40^ were not offered us clearly and the regimen of IC or CCRT were slightly different across-studies might have influenced the efficacy and toxicity of treatment. Fourthly, in the Haddad *et al.* trial^20^, for patients in IC followed by CCRT arm were divided into two groups receiving two different regimens of RT and CCRT, which might cause bias for results. Fifthly, the method of most included trials was unclear, especially in the domain of “allocation concealment and blinding”, which might affect the overall methodological quality of included trials. Only one trial reported allocation concealment in five RCTs^18^. Lastly, the funnel plot analysis showed a potential publication bias from the included trials, which might because of small amount of trials in our meta-analysis, and also one tiral^23^ was actually known that was not available.

In conclusion, on the basis of our meta-analysis, IC followed by CCRT for patients with LA-SCCHN was not statistically significant superior to CCRT alone in OS, PFS, ORR or LRR, but could increase risks of grade 3–4 febrile neutropenia and leukopenia. In contrast, IC followed by CCRT could decrease the incidences of DMR and improve the rates of CR. The current studies do not support the use of IC followed by CCRT over CCRT alone, and the further positioning of IC followed by CCRT as standard treatment for LA-SCCHN will come from more RCTs directly comparing IC followed by CCRT with CCRT alone.

## Methods

### Literature search strategy and identification of eligible trials

Literature searches were carried out in PubMed, EMBASE, Cochrane Library and Chinese Biology Medicine (CBM) from inception to 23 November 2014 with no language restriction by 2 authors. Furthermore, the electronic literature searches were reinforced with manual searches for reference lists of all retrieved articles. In addition, all relevant conference databases that provided grey literature were also searched. Searches included the terms “head and neck cancer”, “head and neck neoplasm*”, “head and neck carcinoma”, “induction chemotherap*”, “neoadjuvant chemotherap*”, “drug therap*”, “chemoradiotherap*”, “concurrent chemoradiotherap*”, “concomitant chemoradiotherap*, “synchronous chemoradiotherap*”, “radiochemotherap*”, “concurrent radiochemotherap*”, “concomitant radiochemotherap*”, “random*”, “randomized controlled trial”. The literature search strategies for each database were reported in the supplementary data.

We included studies that if they met the following criteria: (1) Participating patients were eligible if they had histologically proven stage III-IVM0 SCCHN. (2) Studies combined therapy with IC followed by CCRT versus CCRT alone. (3) RCTs. (4) Reported hazard ratios (HRs) with 95% confidence intervals (CIs) for overall survival (OS) and/or progression-free survival (PFS), or data to calculate these. However, studies were excluded if they were literature published repeatedly, any review, comment, letter, case report, meeting abstracts, trial protocol, animal study, or preliminary result.

### Selection of Literature

Each of the titles and abstracts based on the eligibility criteria were analysed by two reviewers (L.J.Z. and N.J.) independently. Studies were analysed for independent full-text review by the same two reviewers if they could not be excluded from our study based on the titles and abstracts. Any disagreements between reviewers during the selection course were resolved by consensus with the third reviewer (Y.X.S.).

### Data extraction and quality assessment

Data on study year, author, country, study design, sample size, mean/median age (year), male/female, inclusion period, complete treatment, stage, treatment protocol, and major clinical end points (OS, PFS, ORR, CR, DMR, LRR and toxicity) were extracted from all eligible sources by two independent reviewers (L.J.Z. and N.J.). The quality was evaluated by two reviewers independently according to the Cochrane Handbook for Systematic Reviews of Interventions Version 5.1.0 (available from www.cochrane handbook.org). We extracted and examined the random sequence generation, allocation concealment, blinding, incomplete outcome data, and selective outcome reporting from each eligible study. Any disagreements were resolved by consensus with the third reviewer (Y.X.S.). According to these criteria, studies were broadly subdivided into the following 3 categories: A. All quality criteria met: low risk of bias. B. One or more of the quality criteria only partly met: moderate risk of bias. C. One or more criteria not met: high risk of bias.

### Statistical analysis

The primary outcomes were OS and PFS, defined as the time from date of randomization to death from any cause and the time from date of randomization to disease progression or death from any cause without progression whichever occurred first. Results regarding OS and PFS were expressed as hazard ratios (HRs) with 95% confidence intervals (CIs), which could be calculated from the number of observed minus the number of estimated death (O-E) and its variance^41^. Kaplan–Meier curves were read by Engauge Digitizer version 4.1 (free software downloaded from http://sourceforge.net/projects/digitizer/). Results for locoregional recurrence rate (LRR), distant metastasis rate (DMR), overall response rate (ORR), complete response (CR), haematological and non-haematological adverse events were expressed as relative risks (RRs) ratio with 95% CIs using the Mantel–Haenszel method^42^.

Statistical heterogeneity among trials was evaluated by χ^2^ test with a significant level at P < 0.1 and quantified with I^2^ statistic. The fixed-effects model was used if heterogeneity test showed no statistical significance (I^2^ < 50%; P > 0.1). Otherwise we adopted the random-effects model. Sensitivity analysis was conducted to evaluate the stability of the results.

All analyses were conducted in Review Manager version 5.2 (Revman, the Cochrane Collaboration, Oxford, England). A two-sided P-value of <0.05 was considered significant for all analyses except heterogeneity tests.

## Additional Information

**How to cite this article**: Zhang, L. *et al.* Induction chemotherapy with concurrent chemoradiotherapy versus concurrent chemoradiotherapy for locally advanced squamous cell carcinoma of head and neck: a meta-analysis. *Sci. Rep.* **5**, 10798; doi: 10.1038/srep10798 (2015).

## Supplementary Material

Supplementary Information

## Figures and Tables

**Table 1:** **Basic Information of Included Studies.**

**Study**	**Country**	**Study Design**	**Sample Size IC+CCRT/CCRT**	**Mean/median Gge (year) IC+CCRT/CCRT**	**Male/Female IC+CCRT CCRT**	**Inclusion Period**	**Complete Treatment IC+CCRT/CCRT**	**Stage**	**Radiotherapy**	**IC**	**CC**	**Outcomes**
Paccagnella A *et al.* (2010)^18^	Italy	multicenter study	50/51	58/60	46/4 38/13	2003.1-2006.1	46/47	UICC stage III-IVM0	primary tumor: 70 Gy (2 Gy/day, 5 days/week for 7weeks) neck: >60 Gy (2 Gy/day, 5 days/week for 5 weeks).	TPF: docetaxel 75 mg/m^2^ day1, cisplatin 80 mg/m^2^ day 1, 5-fluorouracil 800 mg/m^2^/ day1–4. (every 3 weeks for 3 cycles)	Cisplatin 20 mg/m^2^ day1–4, 5-fluorouracil 800 mg/m^2^ day1–4 (weeks 1 and 6)	OS, PFS, CR,ORR, Toxicity
Chen *et al.* (2011)^19^	China	single-institution study	60/60	55.1/56.5	48/12 46/14	2005.1-2007.6	NR	UICC stage III-IVM0	primary tumor: 66-74 Gy (2 Gy/f × 5 f/week) lymph node(+): 66-70 Gy lymph node(-): 50-54 Gy	Paclitaxel 135-150 mg/m^2^ day1, cisplatin 75–100 mg/m^2^ day1 (every 3 weeks for 2 cycles)	Paclitaxel 135-150 mg/m^2^ day1, cisplatin 75–100 mg/m^2^day1,day22, day43	OS, PFS, CR, PR, SD, PD, ORR, DMR, LRR, Toxicity
Haddad R *et al.* (2013)^20^	USA	multicenter study	70/75	55/54	64/6 63/12	2004.8-2008.12	56/66	AJCC stage III– IVM0	A1: 72 Gy (1.8/1.5 Gy/f × 5 f/week for over 6 weeks) A2: 70 Gy (2.0 Gy/f × 5 f/week for over 7 weeks) B: 72 Gy (1.8/1.5 Gy/f × 5 f/week for over 6 weeks)	TPF: docetaxel 75 mg/m^2^ day1, cisplatin 100 mg/m^2^ day1, fluorouracil 1000 mg/m^2^ day1–4. (every 3 weeks for 3 cycles)	A1: docetaxel 20 mg/m^2^ (weekly for 4 weeks);A2: weekly carboplatin area under the curve (AUC) 1.5 for 7 weeks as per the TAX 324 study^29^; B: cisplatin 100 mg/m^2^ day1,day22	OS, PFS, DMR, LRR, Toxicity
Cohen EE *et al.* (2014)^21^	USA	multicenter study	138/135	56.7/56.9	113/25 118/17	2004.12-2009.5	122/130	AJCC stage IVM0	3DCRT/IMRT: 0.15 Gy /f × 2 f/day every other week Total: 74–75 Gy High/Low -risk microscopic: 54 Gy/39 Gy spinal cord: 40 Gy (3DCRT, 45 Gy (IMRT)	TPF: docetaxel 75 mg/m^2^ day1, cisplatin 75 mg/m^2^ day1, fluorouracil 750 mg/m^2^ day1–5 (every 3 weeks for 2 cycles)	Docetaxel 20 mg/m^2^ and increasing by 5 mg/m^2^ until 30 mg/m^2^, fluorouracil 600 mg/m^2^/ day1–5, hydroxyurea 500 mg PO q12h × 6 days (weeks 1 and 5)	OS, DFFS, RFS, ORR, CR, PR, SD, PD, DMR, LRR, Toxicity
Hitt R *et al.* (2014)^22^	Spain	multicenter study	155,156/128	58.1/56.5	145/10 115/13	2002.12-2007.5	137/107	NR	total tumor: 70 Gy (1.8–2.0 Gy/f × 5 f/week ) lymph node: 50 Gy (1.8–2.0 Gy/f × 5 f/week )	TPF: docetaxel 75 mg/m^2^ day1, cisplatin 75 mg/m^2^ day1, 5-fluorouracil 750 mg/m^2^ day1–5. (every 3 weeks for 3 cycles) PF: cisplatin 100 mg/m^2^ day1, 5-fluorouracil 1000 mg/m^2^ day1–5. (every 3 weeks for 3 cycles)	Cisplatin 100 mg/m^2^ (day1, 22 and 43)	OS, PFS, TTF, LRC, Toxicity

Abbreviations: OS: Overall survival, CR: Complete response, RFS: Recurrence-free survival, PFS: Progression-free survival, PR: Partial response, TTF: Time-to-treatment failure, DFFS: Distant failure–free survival, ORR: Overall response rate, LRC: Locoregional control, DMR: Distant metastasis rate, SD: Stable disease, NR: Not report, LRR: Locoregional recurrence rate, PD: Progerssive disease, IMRT: Intensity-modulated radiation therapy, 3DCRT: Three-dimensional conformal radiation therapy.

**Table 2:** Quality of the five included trials.

**Trial**	**Random sequence generation**	**Allocation concealment**	**Blinding of participants and personnel**	**Blinding of outcome assessment**	**Incomplete outcome data**	**Selective reporting**	**Other bias**	**Rank**
Paccagnella *et al.* 2010^18^	Yes	Yes	Unclear	Yes	Yes	Yes	Yes	B
Chen *et al.* 2011^19^	Yes	Unclear	Unclear	Unclear	Yes	Yes	Yes	B
Haddad *et. al.* 2013^20^	Yes	No	No	No	Yes	Yes	No	C
Cohen *et al.* 2014^21^	Yes	Unclear	No	No	Yes	Yes	Yes	C
Hitt *et al.* 2014^22^	Yes	Unclear	Unclear	Unclear	Yes	Yes	Yes	B

**Table 3:** Haematological and non-haematological adverse events of grade 3–4 during IC period.

**Adverse events**	**Paccagnella** ***et al.*** **2010**^18^	**Chen** ***et al*****. 2011**^19^	**Haddad** ***et al.*** **2013**^20^	**Cohen** ***et al*****. 2014**^21^	**Hitt** ***et al*****. 2014**^22^	**Incidence (%)**
Haematologic						
Anemia	NR	NR	NR	1/136	4/153	1.73%
Thrombocytopenia	NR	NR	NR	4/136	5/153	3.11%
Neutropenia	NR	NR	NR	15/136	29/153	15.22%
Febrile neutropenia	NR	NR	NR	NR	26/153	16.99%
Leukopenia	NR	NR	NR	38/136	24/153	21.45%
Non-haematologic						
Fatigue	NR	NR	NR	10/136	16/153	9.00%
Nausea/Vomiting	NR	NR	NR	8/136	15/153	7.96%
Mucositis	NR	NR	NR	21/136	14/153	12.11%
Pain	NR	NR	NR	2/136	NR	1.47%
Dysphagia	NR	NR	NR	4/136	2/153	2.08%
Anorexia	NR	NR	NR	10/136	NR	7.35%

NR:Not report.

**Table 4:** Haematological and non-haematological adverse events of grade 3–4 during CCRT period.

**Adverse events**	**Paccagnella** ***et al*****. 2010**^18^	**Chen** ***et al*****. 2011**^19^	**Haddad** ***et al.*** **2013**^20^	**Cohen** ***et al*****. 2014**^21^	**Hitt** ***et al*****. 2014**^22^	**Total**	**Risk ratios [95% CI]**	**P-values**
Haematologic								
Anemia	2/43 vs 0/49	3/60 vs 1/60	NR	8/124 vs 4/133	5/106 vs 5/118	18/333 vs 10/360	1.91 [0.91, 4.02]	0.09
Thrombocytopenia	2/43 vs 2/49	3/60 vs 3/60	NR	4/124 vs 2/133	12/106 vs 5/118	21/333 vs 12/360	1.90 [0.95, 3.79]	0.07
Neutropenia	2/43 vs 4/49	5/60 vs 6/60	NR	4/124 vs 2/133	32/106 vs 24/118	43/333 vs 36/360	1.31 [0.88, 1.95]	0.19
Febrile neutropenia	0/43 vs 0/49	NR	16/70 vs 1/75	NR	5/106 vs 1/118	5/149 vs 1/167	11.41 [2.71, 48.03]	0.0009*
Leukopenia	3/43 vs 6/49	7/60 vs 8/60	NR	32/124 vs 15/133	16/106 vs 14/118	57/333 vs 43/360	1.46 [1.01, 2.10]	0.04*
Non-haematologic								
Fatigue	1/43 vs 4/49	NR	NR	6/124 vs 4/133	7/106 vs 4/118	14/273 vs 12/300	1.29 [0.60, 2.74]	0.51
Nausea/Vomiting	0/43 vs 0/49	0/60 vs 0/60	NR	11/124 vs 9/133	8/106 vs 16/118	19/333 vs 25/360	0.85 [0.37, 1.96]	0.70
Mucositis	12/43 vs 18/49	19/60 vs 18/60	33/70 vs 12/75	121/124 vs 119/133	52/106 vs 39/118	237/403 vs 206/435	1.30 [0.86, 1.97]	0.22
Dermatitis	8/43 vs 6/49	10/60 vs 9/60	NR	22/124 vs 32/133	NR	40/227 vs 47/242	0.91 [0.62, 1.33]	0.62
Pain	4/43 vs 5/49	NR	2/70 vs 9/75	13/124 vs 8/133	NR	19/237 vs 22/257	1.42 [0.71, 2.86]	0.74
Dysphagia	9/43 vs 10/49	NR	NR	15/124 vs 20/133	9/106 vs 5/118	33/273 vs 35/300	1.04 [0.67, 1.61]	0.87
Weight loss	2/43 vs 1/49	3/60 vs 2/60	NR	2/124 vs 5/133	NR	7/227 vs 8/242	0.93 [0.34, 2.52]	0.88

NR:Not report Vs:Versus (IC + CCRT vs CCRT) * P < 0.05.
