# Supplementary material for: Induction chemotherapy with concurrent chemoradiotherapy versus concurrent chemoradiotherapy for locally advanced squamous cell carcinoma of head and neck: a meta-analysis
Source: Sci Rep. 2015 Jun 4;5:10798. doi: 10.1038/srep10798 (PMC4455182; doi:10.1038/srep10798)
Supplement: Supplementary Information [file srep10798-s1.pdf]

Induction chemotherapy with concurrent chemoradiotherapy versus concurrent chemoradiotherapy for locally advanced squamous cell carcinoma of head and neck: a meta-analysis.

Lijuan Zhang+, Nan Jiang+, Yuexian Shi, Shipeng Li, Peiguo Wang* & Yue Zhao*

Corresponding Authors: Yue Zhao & Peiguo Wang

Supplementary data

Appendix 1. PubMed search strategy(N=163)

#1 “head and neck cancer”.ti,ab.

#2“head and neck neoplasm*”.ti,ab.

#3“head and neck carcinoma”.ti,ab.

#4 “head and neck neoplasms”.[Mesh]

#5#1OR#2OR#3 OR#4

#6 “induction chemotherap*”.ti,ab.

#7“neoadjuvant chemotherap*”.ti,ab.

#8 “drug therap*”.ti,ab.

#9“induction chemotherapy”.[Mesh]

#10#6OR#7OR#8OR#9

#11“chemoradiotherap*”.ti,ab.

#12“concurrent chemoradiotherap*”.ti,ab.

#13“concomitant chemoradiotherap*”.ti,ab.

#14“synchronous chemoradiotherap*”.ti,ab.

#15“radiochemotherap*”.ti,ab.

#16“concurrent radiochemotherap*” .ti,ab.

#17“concomitant radiochemotherap*” .ti,ab.

#18“chemoradiotherapy”.[Mesh]

#19#11OR#12OR#13OR#14OR#15OR#16OR#17OR#18

#20#5AND#10AND#19

#21“random*”.ti,ab.

#22“randomized controlled trial[publication type]”OR“randomized controlled trials as topic”.[Mesh]

#23#21OR#22

#24#20AND#23

Appendix 2. EMBASE search strategy(N=220)

#1 ' head and neck cancer '.ti,ab.

#2 ' head and neck neoplasm*'.ti,ab.

#3 ' head and neck carcinoma '.ti,ab.

#4 ' head and neck cancer'/exp

#5 ' head and neck tumor'/exp

#6#1OR#2OR#3 OR#4OR#5

#7 ' induction chemotherap*'.ti,ab.

#8' neoadjuvant chemotherap*'.ti,ab.

#9' drug therap*'.ti,ab.

#10' induction chemotherapy'/exp

#11'adjuvant chemotherapy'/exp

#12 #7OR#8OR#9OR#10OR#11

#13' chemoradiotherap*'.ti,ab.

#14' concurrent chemoradiotherap*'.ti,ab.

#15' concomitant chemoradiotherap*'.ti,ab.

#16' synchronous chemoradiotherap*'.ti,ab.

#17' radiochemotherap*'.ti,ab.

#18' concurrent radiochemotherap*'.ti,ab.

#19' concomitant radiochemotherap*'.ti,ab.

#20'chemoradiotherapy'/exp

#21'adjuvant chemoradiotherapy'/exp

#22 #13OR#14OR#15OR#16OR#17OR#18OR#19OR#20OR#21

#23' random*'.ti,ab.

#24' randomized controlled trial '/exp

#25' randomized controlled trial(topic) '/exp

#26 #23OR#24OR#25

#27#6AND#12AND#22AND#26

Appendix 3. CENTRAL search strategy(N=78)

#1 “head and neck cancer”.ti,ab,kw OR “head and neck neoplasm*”.ti,ab,kw OR “head and neck carcinoma”.ti,ab,kw.(Word variations have been searched)

#2 MeSH descriptor.[head and neck neoplasms] explode all trees

#3 #1OR#2

#4 “induction chemotherap*”.ti,ab,kw OR “neoadjuvant chemotherap*”.ti,ab,kw OR “drug

therap*”.ti,ab,kw.(Word variations have been searched)

#5 MeSH descriptor. [induction chemotherapy] explode all trees

#6 #4OR#5

#7“chemoradiotherap*”.ti,ab,kw OR “concurrent chemoradiotherap*”.ti,ab,kw OR “concomitant chemoradiotherap*”.ti,ab,kw OR “synchronous chemoradiotherap*”.ti,ab,kw OR “radiochemotherap*”.ti,ab,kw OR “concurrent radiochemotherap*” .ti,ab,kw OR “concomitant radiochemotherap*” .ti,ab,kw. (Word variations have been searched)

#8 MeSH descriptor. [chemoradiotherapy] explode all trees

#9 #7OR#8

#10“random*”.ti,ab,kw.(Word variations have been searched)

#11 MeSH descriptor. [randomized controlled trials as topic] explode all trees

#12 MeSH descriptor. [randomized controlled trial] explode all trees

#13 #10OR#11OR#12

#14 #3AND#6AND#9AND#13
